# Binding of cellulose binding modules reveal differences between cellulose substrates

**DOI:** 10.1038/srep35358

**Published:** 2016-10-17

**Authors:** Suvi Arola, Markus B. Linder

**Affiliations:** 1 School of Science, Aalto University, P. O. Box 11100, FI-00076, Aalto, Finland; 2 School of Chemical Technology, Aalto University, P.O. Box 16100, FI-00076, Aalto, Finland; 3 VTT, Technical Research Centre of Finland, Bio and process technology, P.O.Box 1000, FIN–02044 VTT, Finland

## Abstract

The interaction between cellulase enzymes and their substrates is of central importance to several technological and scientific challenges. Here we report that the binding of cellulose binding modules (CBM) from *Trichoderma reesei* cellulases Cel6A and Cel7A show a major difference in how they interact with substrates originating from wood compared to bacterial cellulose. We found that the CBM from *Tr*Cel7A recognizes the two substrates differently and as a consequence shows an unexpected way of binding. We show that the substrate has a large impact on the exchange rate of the studied CBM, and moreover, CBM-*Tr*Cel7A seems to have an additional mode of binding on wood derived cellulose but not on cellulose originating from bacterial source. This mode is not seen in double CBM (DCBM) constructs comprising both CBM-*Tr*Cel7A and CBM-*Tr*Cel6A. The linker length of DCBMs affects the binding properties, and slows down the exchange rates of the proteins and thus, can be used to analyze the differences between the single CBM. These results have impact on the cellulase research and offer new understanding on how these industrially relevant enzymes act.

Carbohydrate binding modules (CBMs) are found in a large variety of proteins that are active in one way or another on plant cell walls. These are mainly cellulases but also for example xylanases and mannanases^1^,^2^,^3^,^4^. There are several examples of convergent evolution leading to several different protein families with similar function^5^. Especially in bacteria these families are very diverse^6^. CBMs can show affinity for chitin and are also found as parts of chitin active proteins^7^. In this work we focus on the type of CBMs that are found in fungi and are classified as family^1^,^2^,^8^,^9^. These have been the subject of much investigation because of the efficiency and importance of fungal enzyme systems for degrading cellulosic material both as a part of the ecosystem and also increasingly for technical applications^10^,^11^. These fungal CBMs have a compact structure comprising about 35 amino acids^12^. The structure has a stable cysteine knot fold that is also found in other small adhesion proteins such as conotoxins^13^. Cysteine knots are stabilized by disulfides and in the case of family 1 CBMs two or three of disulfide bridges are found. In the structure of the CBMs there is a distinctive arrangement of three aromatic residues that have been shown to interact with the cellulose surface. The interaction between aromatics and pyranose rings is widely observed also elsewhere, and it has been shown that by changing the character of the aromatic residues also the binding characteristics can be changed^14^. In addition to these pi-electron interactions, hydrogen bonding is involved in forming affinity and specificity between protein and cellulose^12^. The binding is highly specific and shows even selectivity for the different crystalline faces of cellulose^15^,^16^.

The CBMs have proved to play an essential role for how enzymes function. There are effects on both substrate recognition and catalytic activities^16^,^17^. The most straightforward explanation of CBM function is to guide the enzyme towards the substrate and increase local concentrations on surfaces, but in many cases the role has been found to be more subtle^2^. For example the exchange rate of cellulases from their substrates is greatly affected by the presence of a CBM^17^

In this work we set out to gain understanding of both cellulose as a substrate, and the functional role of CBMs in enzymes acting on cellulosic materials. It is already known that linking two CBMs together does affect the overall interaction of the proteins and substrate to a very large extent and that this two-domain interaction can to some extent explain the architecture of cellulose degrading enzymes in general^7^. The thermodynamic principles behind such cooperativity are well known^18^ and depend on geometric constraints and the general architecture of the complexes that are formed. We hypothesized that making linkers with different lengths between two CBMs could reveal how binding sites are located on the cellulose surface and how the linking of binding modules changes the dynamics of interactions. Using a set of differently designed molecules, different sources of cellulose, and accurate measurement techniques enabling the study of binding kinetics allowed a new set of tools to investigate cellulose as complex structure and the structure-function relationship of the enzymes acting on it.

## Results

### Analysis of proteins

Double CBMs (DCBMs) with different linker lengths were produced as HFBI hydrophobin fusions (HFBI-DCBM) to aid purification (DCBM sequences shown in Supplementary Figure S1) as described previously^19^. The linkers were 12, 24 and 48 amino acids long and the proteins were named accordingly DCBM-12, −24 and −48. The DCBM proteins were obtained by trypsin cleavage of the corresponding HFBI-DCBM. The CBM-*Tr*Cel7A and CBM-*Tr*Cel6A were obtained by papain cleavage of HFBI-DCBM-12. Amino acid analysis (AAA) of the fractions from RP-HPLC run after papain cleavages showed the identity of the two fractions; i.e. which of them CBM-*Tr*Cel7A was and which CBM-*Tr*Cel6A.

Matrix-assisted laser desorption/ionization – time of flight mass spectrometry (MALDI-TOF MS) was used to verify the size of all the proteins and the extent of glycosylation of the linker regions. According to MALDI-TOF results DCBM-24 contained14–26 glycan groups and DCBM-48 approximately 28–50 glycan groups. Both linkers (DCBM-24 and −48) contain multiple *O*-glycosylation sites (threonine (T) and serine (S), Supplementary Figure S1). The glycosylation patterns were heterogenic and showed a broad peak at m/z ~12730–14500 for DCBM-24 and m/z ~18400–20730 for DCBM-48. DCBM-12 did not show similar heterogenic glycosylation as DCBM-24 and −48. DCBM-12 had a major peak at m/z 9039 and two minor peaks at m/z 9265 and 9446.

The single CBMs cleaved from HFBI-DCBM-12 by papain digestion did not show glycosylation in MALDI-TOF. The AAA results showed about 0,5% of glucosamine in the sample, which corresponds to 1 GlcNAc per 200 amino acids for both CBM samples. There were no peaks with differences by m/z 162 (hexose) or m/z 203 (GlcNAc). The main peak for CBM-*Tr*Cel6A was at m/z 5414 with minor peaks at m/z 5015, 5170, 5214, 1257, 5313, 5370, 5501, 5572, 5659, and 5743. CBM-*Tr*Cel7A main peak was at m/z 3850 with minor peaks at m/z 3763, 3832, 3952, 4051, and 4251. For the major and minor peaks Na-adducts were seen.

### Binding isotherms

**Figure 1:**
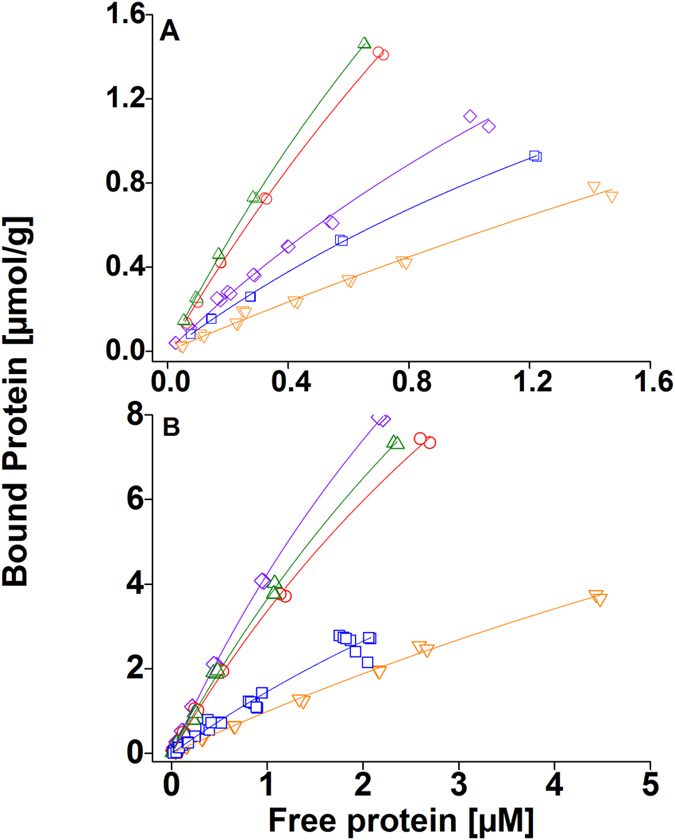
The initial slopes of the binding isotherms for CBM-Cel7A, CBM-Cel6A, DCBM-12, DCBM-24, and DCBM-48 on CNF and BMCC. (**A**) CBM-Cel7A and CBM-Cel6A, DCBM-12, DCBM-24, and DCBM-48 on BMCC, (**B**) CBM-Cel7A and CBM-Cel6A, DCBM-12, DCBM-24, and DCBM-48 on CNF. Violet diamond: CBM-CeI7A, orange triangle: CBM-Cel6A, red circle: DCBM-12, green triangle: DCBM-24, and blue square: DCBM-48.

We performed binding studies of all five CBM constructs with varying concentrations on two different substrates, namely cellulose nanofibrils (CNF) originating from hardwood and bacterial microcrystalline cellulose (BMCC). Figure 1 shows the initial slopes of the binding isotherms for CBMs and DCBMs on a) BMCC, and b) CNF. The corresponding partitioning coefficients, K_r_, shown in Table 1 can be calculated from the dissociation constant, K_d_, and the maximum binging capacities, B_max_, using equation (1), where B is the amount of bound protein and Free is the amount of unbound protein.


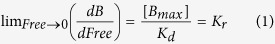


The K_d_ and and B_max_ values obtained from curve-fitting and used to calculate the K_r_-values are listed in Supplementary Table S5. On BMCC DCBM-12 and DCBM-24 constructs have a significantly higher K_r_-value compared to single CBMs and DCBM-48. On CNF the K_r_-values for the DCBMs followed the same order as on BMCC; K_r_ (DCBM-24) > K_r_ (DCBM-12) > K_r_ (DCBM-48). The K_r_-value of CBM-*Tr*Cel7A on CNF was higher than for any other protein and the K_r_ for CBM-*Tr*Cel6A was the lowest.

To compare binding on the two substrates, the ratio of K_r_-values (K_r_^CNF^/K_r_^BMCC^) was calculated (Table 2). This ratio was the same for all other proteins, (about 1.5), except for CBM-*Tr*Cel7A for which it was 3.6.

**Figure 2:**
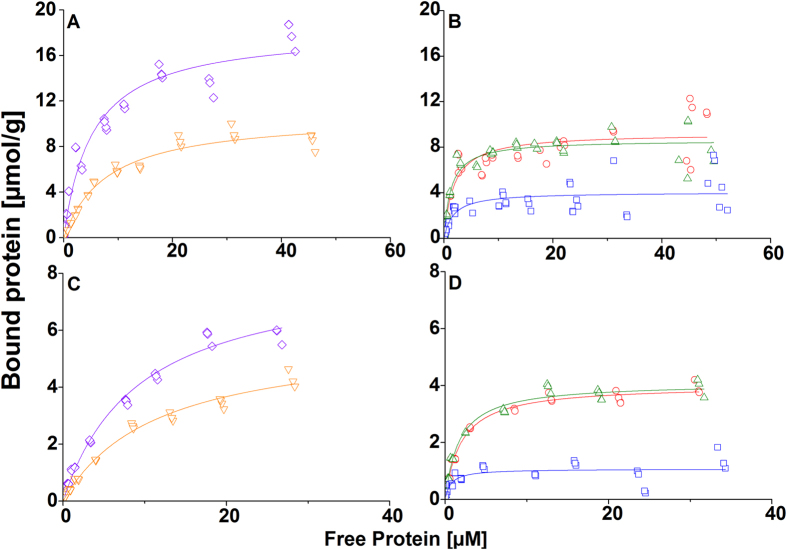
Binding isotherms for CBM-Cel7A, CBM-Cel6A, DCBM-12, DCBM-24, and DCBM-48 with high protein concentrations on CNF and BMCC. (**A**) CBM-Cel7A and CBM-Cel6A on CNF, (**B**) DCBM-12, −24, −48 on CNF, (**C**) CBM-Cel7A and CBM-Cel6A on BMCC, and (**D**) DCBM-12, −24, −48 on BMCC. Violet diamond: CBM-CeI7A, orange triangle: CBM-Cel6A, red circle: DCBM-12, green triangle: DCBM-24, and blue square: DCBM-48.

Figure 2 presents the binding isotherms of all five proteins for both substrates with large protein concentrations (up to 50 μM free protein concentration). The semi-logarithmic plots of the same data are presented in Supplementary Figure S2 to illustrate the saturation of the binding. The B_max_ obtained from these data are presented in Table 3.

### The Gibbs free energy of binding, ∆G

In order to identify differences in the binding energies of the proteins that could account for the different behavior of CBM-*Tr*Cel7A on CNF, we calculated the ΔG-values for all five proteins on both substrates using the B_max_-values obtained from the data presented in Fig. 2 (Supplementary Figure S2) and the K_r_-values presented in Table 1 using equation (2).


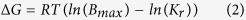


For the binding of DCBM-48 on both substrates the maximum binding capacity was clearly reached (Fig. 2b,d and Supplementary Figure S2b,d). Thus, the B_max_-values could be used as such for the ΔG calculations. For the other proteins the actual B_max_ was not reached in our experiments (Fig. 2 and Supplementary Figure S2). In order to calculate an estimated range of the ΔG-values for these proteins, we used the K_r_-values (Table 1) and a range of the B_max_-values in equation (2). The B_max_-value range used was B_max_, _low_, B_max_, _intermediate_, and B_max_, _high_. A schematic illustration of the three B_max_ –values are presented in Supplementary Figure S3 with the binding isotherm for CBM-*Tr*Cel7A on CNF. Table 4 summarizes the ΔG-values for the different proteins on both substrates.

### Exchange rates of CBMs and DCBMs on CNF and BMCC

**Figure 3:**
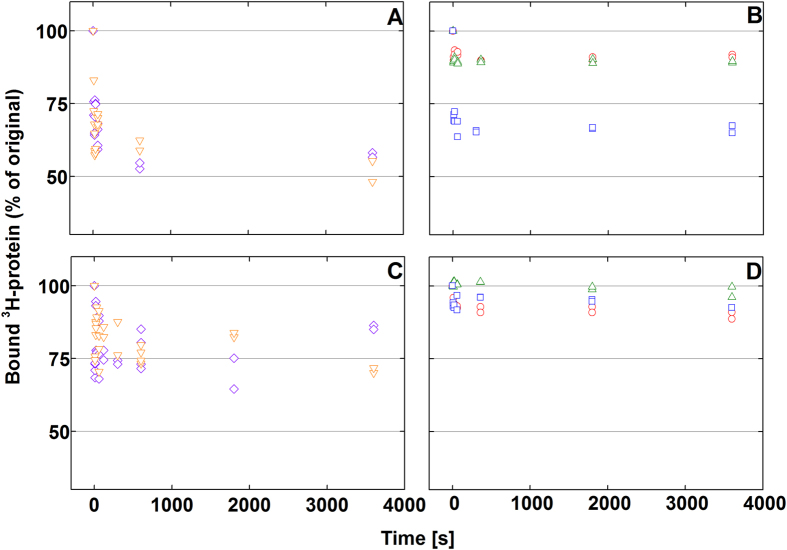
The exchange of ^3^H-labelled proteins from substrate surface with the non-labelled protein in the solution during time. (**A**) Exchange of ^3^H-labelled CBM-Cel7A and CBM-Cel6A from BMCC surface, (**B**) Exchange of ^3^H-labelled DCBM-12, DCBM-24, and DCBM-48 from BMCC surface, (**C**) Exchange of ^3^H-labelled CBM-Cel7A and CBM-Cel6A from CNF surface, and (**D**) Exchange of ^3^H-labelled DCBM-12, DCBM-24, and DCBM-48 from CNF surface. Violet diamond: CBM-CeI7A, orange triangle: CBM-Cel6A, red circle: DCBM-12, green triangle: DCBM-24, and blue square: DCBM-48.

Next, to investigate the exchange of DCBMs and CBMs on both substrate surfaces, we performed a series of experiments measuring the exchange rate at steady-state between ^3^H-labelled proteins and unlabeled proteins. The results presented in Fig. 3a show that exchange between adsorbed and free CBM-*Tr*Cel7A and CBM-*Tr*Cel6A readily occurs on BMCC surface, reaching 50% of labelled protein on the surface from the original amount. The rates of exchange are fast. The exchange occurs during tens to hundreds of seconds, which is in line with previous results gained for CBM-*Tr*Cel7A on BMCC^20^. Only partial exchange was detected in the case of DCBMs on BMCC; ~10% of DCBM-12 and −24, and ~30% of DCBM-48 exchange (Fig. 3b). Approximately 20–25% of the single CBMs (Fig. 3c) and 5–10% of the DCBMs (Fig. 3d) exchange on the CNF surface.

### Nanoscale effect of the CNF substrate

CNF is much finer in structure due to its nanoscale scale size than commonly used substrates for cellulase studies such as microcrystallinen cellulose (MCC) or BMCC. To test if the nanoscale structure of the substrate affect the binding properties of the proteins we performed binding studies on the pulp material that the CNF was prepared from. The initial slopes of the binding isotherms and the corresponding K_r_-values are shown in Supplementary Figure S4. The order of binding on pulp was the same as for CNF; CBM-*Tr*Cel7A has the steepest initial slope and thus the largest partitioning coefficient followed by the DCBMs and CBM-*Tr*Cel6A. The K_r_-values are very close to those obtained for CNF and thus the binding mode or mechanism seems to be the same as for CNF.

### Xylanase assay for CBM-*Tr*Cel7A

The CNF that was used for the experiments was found to contain roughly 27% xylan by total enzymatic hydrolysis and sugar content analysis. Of this, 30% can be specifically hydrolyzed by pI9 xylanase from *T. reesei*, which corresponds to about 10% loss of the total mass^21^. To test how the xylan on CNF affects the CBM-*Tr*Cel7A binding we used the pI9 xylanase to hydrolyze xylan from CNF and tested how the treatment affected the binding of CBM-*Tr*Cel7A on CNF. We used a fixed concentration of protein and xylanase treated CNF, and quantified the amount of free protein from the reaction mixture using tritium labeled CBM-*Tr*Cel7A. As a control for the experiment we used CNF that was not treated with xylanase. All reactions were carried out in three replicates. The results showed that the binding of CBM-*Tr*Cel7A increased about 20% on the xylanase treated CNF compared to the non-treated CNF.

## Discussion

The results of this study show that the linker length of the DCBMs had a large effect on their binding properties and that the binding properties are greatly affected by the origin of the cellulose substrate. Surprisingly, we found out that CBM-*Tr*Cel7A, had a much higher partitioning coefficient on wood cellulose compared to the other proteins (Table 1), although, based on previous studies^7^ and general knowledge on polyvalent interactions^18^ we anticipated that the DCBMs would have a higher affinity towards cellulosic substrates than the single CBMs. We also saw that in addition to effects on the binding properties the linking of the CBMs together slowed down the exchange of bound protein from the substrate surface regardless of the substrate origin (Fig. 3b,d). However, the substrate origin had an effect on the exchange of the single CBMs (Fig. 3a,c).

The K_r_-values allow comparison of the binding efficiency of the different proteins. But to investigate the difference of protein binding between substrates, BMCC and CNF, we calculated the ratio of K_r_-values for each protein on the two different substrates (Table 2). The ratios of the partitioning coefficients for the DCBMs and CBM-*Tr*Cel6A were about 1.5. This result indicates that these proteins bind to the two substrates in a similar manner, except that CNF has 1.5 times more surface area per weight than BMCC. CBM-*Tr*Cel7A on the other hand had an overall higher K_r_-value on CNF compared to the other proteins, and the ratio of K_r_-values was over two times higher than for the other constructs. From these results we can deduce that the CBM-*Tr*Cel7A has another binding mode or site on CNF that it does not have on BMCC, and which the CBM-*Tr*Cel6A or the DCBMs do not have. Some feature in CNF is available for CBM-*Tr*Cel7A that does not exist in BMCC, yet this feature is not available for the other constructs. It is especially notable that the synergies between modules seen for the DCBM-12 and DCBM-24 when binding to BMCC are not seen when the proteins bind to CNF. DCBM-12 and DCBM-24 are thus not able to incorporate a possible extra mode of binding (e.g. conformational changes) or binding site of CBM-*Tr*Cel7A when they bind to CNF, yielding lower K_r_-values compared to CBM-*Tr*Cel7A.

The linker length had an effect on the binding properties of the DCBMs following the same order on both substrates; K_r_(DCBM-24) > K_r_(DCBM-12) > K_r_(DCBM-48). These results indicate that there is an optimum linker length for the DCBMs in respect to maximum binding affinity, close to or equal to 24 amino acids. The positive effect on the affinity of multivalent interactions is well known^18^. In our case, however, it seems to be substrate dependent behavior, which is dictated by unexpected binding characteristics of one of the binding units.

To investigate if the overall higher affinity of CBM-*Tr*Cel7A towards CNF would be caused by a more favorable binding energy we estimated the ΔG-values for the different binding events. In order to conduct this estimation, we first experimentally determined reliable values for maximum binding capacities, B_max_, because the uncertainty of the B_max_ (and hence the k_d_) is large when only using initial parts of the isotherms for fitting. Thus, we evaluated if measuring full binding isotherms, allowing the calculation of binding energies, would be feasible. The uncertainty of B_max_-values is clearly seen by the differences of the estimated values shown in Table S5 compared to the actual measured values shown in Table 3. Data points were collected at very high protein concentrations (around 50 μM). At these concentrations only about 1–10% (DCBM) or 20% (CBM) of the total protein are bound to cellulose. Due to the high overall protein concentrations and low percentage of total protein bound to cellulose it was not feasible to acquire data points at even higher concentrations. A small error (1–2%) in measuring the free protein concentration would result in a large error in bound protein amount (40–50%). The data points are shown in Fig. 2, and as semi-logarithmic plots in Supplementary Figure S2 to illustrate how close to saturation the binding curves are. The B_max_-values obtained from the full binding isotherms are presented in Table 3. From the data it is seen that the DCBM-48 has reached its maximum binding capacity on both substrates, and that the DCBM-12 and −24 are very close to reaching their actual maximum binding capacity. Using the structure of CBM-Cel7a determined by Kraulis *et al.*^22^ we can estimate that one CBM requires at least 1.8 nm * 3 nm surface area of cellulose to bind, that is 5.4 nm^2^. This gives a lower limit for the available surface area to bind of 59 m^2^/g and 27 m^2^/g for CNF and BMCC, respectively. The B_max_-values for DCBM-48 are lower on both substrates compared to the B_max_-values of DCBM-12 and DCBM-24. The single CBMs have generally higher B_max_-values on both substrates compared to the DCBMs, and they have not reached the actual maximum binding capacities. This too low B_max_ can lead to an underestimation of ΔG. The natural linker of *Tr*Cel7A enzyme is 27 amino acids long^23^ and that of *Tr*Cel6A enzyme is 51 amino acids long^24^. The linker length clearly affects both the binding affinity and the binding capacity of the DCBMs and can thus also be a crucial element in controlling the binding affinity and capacity of the cellulases in nature. Igarashi *et al.* have shown that if the packing density of the enzymes on the substrate surface is too high the processivity of the cellulase is hindered^16^. In this respect the double binding module structure of the cellulase could be beneficial not only by concentrating or locating the enzyme on the substrate surface^25^ but also by controlling the amount of enzyme on the substrate surface, and in this way contributing to the hydrolysis efficiency of the enzymes.

The ΔG-values, presented in Table 4, show that the binding energies for DCBMs are generally more negative than those for CBMs (~4–16% on CNF and ~10–17% on BMCC), and thus show that for all DCBMs there is a synergy in binding that comes from the linkage. In this regard it is energetically favorable to link CBMs together as DCBMs. It is also logical when considering the action of cellulase enzymes. However, these results do not explain the higher affinity of CBM-Cel7A on CNF.

Next we studied the exchange of free and bound protein on both substrates by equilibrium exchange experiments. On BMCC both CBMs alone fully exchanged to 50% of the original amount rather quickly (<600 s) and thus showing true reversibility (Fig. 3a) as previously shown for CBM-*Tr*Cel7A on BMCC^26^. On CNF, the single CBMs did not exchange to 50% from the original amount of bound, but only to 20–25% (Fig. 3c) showing that the CBMs experience the two substrates differently.

The DCBMs showed only partial exchange from BMCC (Fig. 3b) and CNF (Fig. 3d), 10–30% from BMCC and 5–10% from CNF. This partial exchange has been previously seen for HFBI-DCBM on CNF^27^. In nature slow processive enzymes such as cellulases could benefit from this reduced amount of protein exchange caused by the linking of two affinity proteins allowing more time for the hydrolysis reaction. It has been shown with high speed atomic force microscopy that *Tr*Cel7A cellulase carrying a mutation in its catalytic domain preventing its binding to the substrate surface and *Tr*Cel7A lacking the CBM have a faster exchange rate and lower activity than the wild type enzyme^17^. In this respect a double affinity module with reduced exchange from the substrate surface seem to be more effective in cellulose hydrolysis than a single affinity module with higher and potentially fully reversible exchange.

To investigate if mechanical processing of pulp to CNF had an effect on the substrate, we determined the initial slope of the binding isotherm on the pulp material used to make the CNF. The partitioning coefficients of the proteins on pulp were very close to those obtained for CNF and that the order of binding was the same (Supplementary Figure S4) showing that CNF processing was not the reason for our results.

We next considered if differences in polysaccharide composition between the two substrates that could explain affect results. BMCC is pure cellulose whereas CNF form birch has, in addition to cellulose, roughly 27% xylan^21^. To try and understand the difference in binding sites available for CBM-*Tr*Cel7A on CNF but not on BMCC, we investigated how the specific hydrolysis of xylan affected the binding. This was done by using a xylanase enzyme to hydrolyze xylan from CNF and to study the binding of CBM-*Tr*Cel7A after the treatment in comparison to non-treated CNF. We saw that the amount of bound CBM-*Tr*Cel7A was increased by approximately 20% after xylanase treatment. From previous studies we know that the xylanase treatment reduces the amount of xylan in CNF by roughly 30%^21^. This indicates that the CBM-*Tr*Cel7A finds more binding sites on the fibril surface after xylanase treatment due to an increase in the amount of exposed cellulose surface. Thus, the higher affinity of CBM-*Tr*Cel7A towards CNF would not be caused by an additional affinity towards xylan that is present in the CNF material. It has also been shown with cellulases, that xylanase enzymes are needed in order for the cellulases to fully hydrolyze CNF and that cellulases alone are not enough to hydrolyze materials that contain hemicelluloses^28^. We showed here that the binding of CBM-*Tr*Cel7A is positively affected by xylan hydrolysis and that at least partly the xylan available for the xylanase is located on the surface of the fibril covering the CBM-*Tr*Cel7A binding sites.

It now seemed evident that the differences in the substrates that are most obvious were not fully explaining the unexpected behavior of CBM-*Tr*Cel7A upon binding to CNF. The features, which are origin dependent in cellulose substrates are their crystalline structure and amorphous to crystalline ratio; wood cellulose crystalline regions compose mainly of cellulose I_β_ and BMCC of cellulose I_α_, and BMCC is more crystalline^29^,^30^,^31^. Finding a way to test the effects of the crystalline structure on the binding properties is tricky, and some methods to analyze the morphological targets of CBMs have been devoleped^25^, but by competition experiments we could see if the two CBMs share binding sites on the two substrates. This on the other hand might reveal differences on the substrate structures. To test if the two CBMs fully or partially compete on binding sites on CNF and BMCC we designed a set of experiments where we tried to see if the binding of the one CBM is affected by the presence of the other. This was examined by competing one labelled CBM with another unlabeled CBM (Experimental in Supplementary Information, data in Supplementary Figure S6). The results showed that the binding of both labeled CBMs were affected by the presence of the unlabeled different CBM in the same way as they were affected by the presence of the unlabeled identical CBM. This suggests that the two CBMs would fully compete on binding sites on both substrates, and that the deviant binding behavior of CBM-*Tr*Cel7A on CNF would be caused by an extra mode of binding rather than an extra binding site recognized only by CBM-*Tr*Cel7A.

The more efficient binding of the CBM-*Tr*Cel7A on CNF could also be caused by *O*-mannosylation of the binding module near the binding site^32^,^33^. Our proteins were produced in *T. reesei*, and the introduction of *O*-glycans could be possible. Yet, we saw no evidence of mannosylation of the CBMs with MALDI-TOF MS and AAA. The main peak of CBM-*Tr*Cel7A at m/z 3850 was very close to the average molecular mass obtained by AAA (3828 Da) and to the theoretical molar mass of the CBM-*Tr*Cel7A calculated from the amino acid sequence (3828 Da). None of the minor peaks matched with hexoses or HexNAc. The AAA results showed only little variation in the amounts of individual amino acids (aa) and the variation was concentrated on the amino acids present in the linker; namely serine and threonine. There were some traces of glucoseamine in the sample (~0.5 mol-%, meaning 1 sugar molecule per 200 amino acids) but this was too low to account for protein glycosylation. Similar findings were made for CBM-*Tr*Cel6A with AAA. However, more variation was seen in the linker region aa amounts compared to CBM-*Tr*Cel7A. This indicates that the linker regions present on both sides of the CBM-*Tr*Cel6A show some heterogeneity due to papain cleavage. In contrast, the CBM-*Tr*Cel7A has only N-terminal flanking linker. There were similar amounts of glucosamine present in the CBM-*Tr*Cel6A sample as was in the CBM-*Tr*Cel7A sample indicating a minor co-purification with the protein, rather than glycosylation. Furthermore, the mass differences in MALDI-TOF MS did not match glycan groups. Our results from AAA and MS data concluded that the single CBMs were not glycosylated, and that the variation of masses with in the samples arouse from the variation in the lengths of the flanking linker regions.

### Conclusions

The work reveals that there is a difference in the way in which CBM-Cel7A interacts with cellulose of different origin compared to CBM-Cel6A. The CBM therefore has a direct effect on the substrate recognition of cellulase and not only on as previously reported, on the location of binding on the cellulose crystal. The results also show that BMCC may not be an ideal substrate for cellulase structure-function studies if the aim is to understand how plant cell walls are degraded. The results also show that substrate recognition is affected by the way in which CBMs are linked. The linkage of CBM-Cel6A to CBM-Cel7A in the DCBD did not give coupled binding energies nor exchange rates in the same way on CNF as on BMCC. This study shows that the CBM-substrate interaction is more complex than previously predicted and highlights in a new way that the choice of model substrates can affect outcomes of cellulase structure function studies.

## Methods

### Cellulose substrates

Cellulose nanofbrils (CNF) were prepared by fluidizing never-dried bleached Birch pulp (kappa number 1; DP 4,700; fines removed (SCAN-M 6:69)). The pulp was washed to the sodium form according to Swerin *et al.*^3^. The washed pulp was disintegrated with a high-pressure fluidizer (Microfluidics M110P, Microfluidics Int. Co., Newton, MA) in 12 passes essentially according to previous reports^31^. No chemical or enzymatic pre-treatment was used prior to disintegration. Bacterial microcrystalline cellulose (BMCC) was prepared from Nata de Coco by several washing and grinding steps.

### Protein production and purification

The DCBMs were produced as HFBI-DCBM fusions in *T. reesei* as described in details in earlier work of the group^19^ (DCBM sequences in Supplementary Figure S1) and the following transformants were used: VTT-D-133335 (HFBI-DCBM-12), VTT-D-133336 (HFBI-DCBM-24), and VTT-D-133337 (HFBI-DCBM-48). The strains were then cultivated in 7 L bioreactors on media containing 50 vol-% spent grain extract, 60 g/L lactose, 1 g/L yeast extract, 4 g/L KH2PO4, 2.8 g/L (NH4)2SO4, 0.6 g/L MgSO4 ∙ 7H2O, 0.8 g/L CaCl2 ∙ 2H2O, 2 ml/L trace solution. The pH was let to drop from 5 to about 3 during cultivation. At 24 h intervals 48 mg pepstatin A and 28 mg soy bean trypsin inhibitors (both from Sigma-Aldrich) were added to the cultures to minimize protein degradation. The culture supernatants were separated from the biomass by filtration through GF/A filters (Whatman). Protein expression levels were analyzed by RP-UPLC and were 0.2 g/L, 0.4 g/L, and 3.0 g/L for HFBI-DCBM-12, −24, and −48, respectively. The proteins were purified using aqueous two phase extraction and reverse-phase high-performance liquid chromatography (RP-HPLC) as described earlier^35^ followed by lyophilization.

### Protein preparation

The fusion proteins were cleaved with trypsin (Promega) in 25mM Tris-HCl – 150mM NaCl buffer pH 7 for 2 hours to yield DCBMs. The trypsin digestion reaction was followed by RP-UPLC using a 2.1 × 100 mm, 1.7 μm, C4 BEH300 prST column and an Acquity I-Class system (Waters, MA, USA). The proteins were eluted by a gradient from 20–60% water to acetonitrile, both containing 0.1% trifluoroacetic acid. Concentrations were determined using standard samples determined by amino acid analysis (Uppsala University, Sweden).

The HFBI-DCBM-12 was cleaved using papain (Sigma), in 0.1 mM Sodium Phosphate buffer pH 7 for o/n in room temperature to yield CBM-*Tr*Cel7A and CBM-*Tr*Cel6A. The identity of CBMs were verified by amino acid analysis and MALDI-TOF MS (Autoflex II).

### Protein labelling

Proteins were labelled with tritium by reductive methylation^7^,^20^,^27^. 1.9 mg of protein was dissolved in 1.9 mL of 0.2 M borate buffer, pH 8.96 and cooled on an ice bath. 13.2 μL of 0.37% formaldehyde and 100 mCi of ^3^H enriched NaBH4 (10 Cimmol^−1^, PerkinElmer) in 150 μL of 0.01 M NaOH were added and reacted for 30 minutes. The reaction was terminated by RP-HPLC. The specific activities were 0.52 Cimmol^−1^, 0.69 Cimmol^−1^ and 1.25 Cimmol^−1^ for DCBM-12, −24 and −48.

### Binding isotherms

Binding studies were done essentially as reported previously^20^,^27^,^36^,^37^. A 100 μM stock solution with 10% ^3^H–labelled proteins in 100 mM sodium acetate buffer (pH 5.0) with 100 mM NaCl and 0.1% BSA was prepared. Dilutions were made with the same buffer. 100–200 μL of each protein solution was mixed with an equal volume of 1–2 g L^−1^ CNF in water (or 1.28 gL^−1^ BMCC) and stirred at ambient temperature with 250 rpm for 1 h. The suspensions were then filtered (Millipore, Millex^®^–GV filter unit, 0.22 μm,) and the free protein was determined by liquid scintillation (PerkinElmer, Tri-Carb 2810TR).

### Xylanase assay

The pI9 xylanase of *T. reesei* was used to hydrolyze xylan from CNF^21^,^38^. The hydrolysis of CNF was carried out for 24h in 45 °C. In a control CNF sample xylanase was ommitted. The hydrolysed and non-hydrolysed CNF was used to study the effect of xylan hydrolysis with CBM-*Tr*Cel7A using a 2.5 μM stock solution of the CBM containing 10% 3H-labelled protein. The reactions were terminated by filtrating.

### Exchange rate assay

Exchange rate experiments were done as previously^26^,^39^ by competition between ^3^H-labeled and unlabeled protein. A 100 μL aliquot of BMCC (1.28 g L-1) or CNF (2 g L-1) was mixed with 100 μL ^3^H-labelled protein. After binding, the same amount of unlabeled protein was added. The concentration of the added protein was the same as that of the free protein in the equilibrium reaction. The experiments were carried out by taking multiple time points from a set of parallel samples.

### Calculations used for partitioning coefficient and Gibb’s free energy difference

Langmuir one-site binding model is written in the equation (3) below.


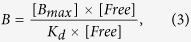


where B_max_ is the maximum binding capacity, K_d_ is the dissociation constant, and [Free] is the concentration of the free protein. When the free protein concentration approaches zero the initial slope of the curve is described by equation (4),


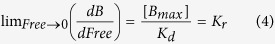


where K_r_ is the partitioning coefficient and describes the relation of adsorption and desorption at very low protein concentrations.

Gibb’s free energy is (equation (5)),


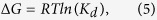


and using equation (1) it can be written as show in equation (6),


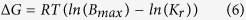


where R is the universal gas constant and T is temperature.

## Additional Information

**How to cite this article**: Arola, S. and Linder, M. B. Binding of cellulose binding modules reveal differences between cellulose substrates. *Sci. Rep.* **6**, 35358; doi: 10.1038/srep35358 (2016).

## Supplementary Material

Supplementary Information

## Figures and Tables

**Table 1:** The partitioning coefficients (K_r_ = B_max_/k_d_) for single and double CBM obtained from the Langmuir isotherm fitted to the data on Fig. 1a,b.

	CBM-Cel7A	CBM-Cel6A	DCBM-12	DCBM-24	DCBM-48
BMCC	1.40 ± 0.46	0.61 ± 0.46	2.47 ± 0.55	2.82 ± 0.45	1.07 ± 0.15
CNF	4.98 ± 0.56	1.05 ± 0.34	3.87 ± 0.75	4.18 ± 0.85	1.67 ± 1.12

**Table 2:** The correlations of partitioning coefficients, K_r_, for single and double CBM between CNF and BMCC calculated from K_r_-values in Table 1.

	CBM-Cel7A	CBM-Cel6A	DCBM-12	DCBM-24	DCBM-48
K_r_^CNF^/K_r_^BMCC^	3.57	1.72	1.56	1.49	1.56

**Table 3:** Binding capacities, B_max_ (μmolg^−1^), for single and double CBM on CNF and BMCC obtained from the data shown in Fig. 2.

	CBM-Cel7A	CBM-Cel6A	DCBM-12	DCBM-24	DCBM-48
BMCC	8.20 ± 0.30	5.78 ± 0.21	4.01 ± 0.07	4.09 ± 0.08	1.07 ± 0.08
CNF	18.24 ± 0.66	10.74 ± 0.37	9.21 ± 0.34	8.61 ± 0.21	4.05 ± 0.23

**Table 4:** Gibbs free energy for the binding, ΔG (kJ/mol), for CBM-Cel7A, CBM-Cel6A, DCBM-12, DCBM-24, and DCBM-48 on CNF and BMCC.

substrate	B_max_*	CBM-Cel7A	CBM-Cel6A	DCBM-12	DCBM-24	DCBM-48
CNF	low	−30.7	−28.2	−31.8	−32.1	−31.7
	inter	−29.0	−26.5	−30.1	−30.4	
	high	−28.0	−25.5	−29.1	−29.4	
BMCC	low	−29.6	−28.4	−32.7	−33.0	−33.9
	inter	−27.9	−26.7	−31.0	−31.3	
	high	−26.9	−25.7	−30.0	−30.3	

^*^B_max_−value for DCBM-48 from data in Fig. 2 is an accurate value, thus it can be used to calculate the binding energy associated with the binding event. The ΔG-values for CBM-Cel7A, CBM-Cel6A, DCBM-12 and DCBM-24 are estimates (see supplementary information for the estimation, Supplementary Fig. 3). B_max,low_ is the value gained from data on Fig. 2, inter is 2x (B_max,low_), and high is 3x(B_max,low_).
