# Supplementary material for: Binding of cellulose binding modules reveal differences between cellulose substrates
Source: Sci Rep. 2016 Oct 17;6:35358. doi: 10.1038/srep35358 (PMC5066208; doi:10.1038/srep35358)
Supplement: Supplementary Information [file srep35358-s1.pdf]

Binding of cellulose binding modules reveal differences between cellulose substrates

Suvi Arola and Markus B. Linder

Supplementary Information

Sequences of the DCBM proteins

DCBM-12
RGPGGQACSSVWGQCGGQNWSGPTCCASGSTCVYSNDYYSQCLPGANPPGTTTTS
TQSHYGQCGGIGYSGPTVCASGTTCQVLNPYYSQCL

DCBM-24
RGPGGQACSSVWGQCGGQNWSGPTCCASGSTCVYSNDYYSQCLPGANPPGTTTTS
QPATTTGSSPGPTQSHYGQCGGIGYSGPTVCASGTTCQVLNPYYSQCL

DCBM-48
RGPGGQACSSVWGQCGGQNWSGPTCCASGSTCVYSNDYYSQCLPGANPPGTTTTS
QPATTTGSSPGPPGANPPGTTTTSQPATTTGSSPGPTQSHYGQCGGIGYSGPTVCASG
TTCQVLNPYYSQCL

Supplementary figure S1. Amino acid sequences of the three different DCBMs that were used in this study. DCBM-12 was used to gain CBM-Cel7A and CBM-Cel6A by papain cleavage. Linker regions are in black, CBM-Cel6A sequence is shown in red, and CBM-Cel7A sequence is shown in green.

Binding isotherms of CBM and DCBM on CNF and BMCC

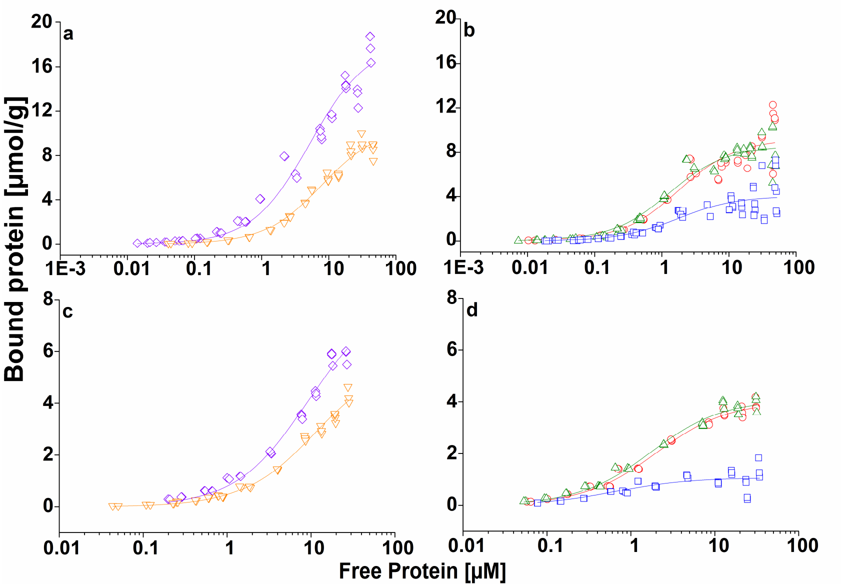

Supplementary figure S2. Semi-logarithmic representation of binding isotherms with high protein concentrations of **a)** CBM-Cel7A, CBM-Cel6A on CNF, **b)** DCBM-12, DCBM-24, and DCBM-48 on CNF, **c)** CBM-Cel7A, CBM-Cel6A on BMCC, **d)** DCBM-12, DCBM-24, and DCBM-48 on BMCC. Violet diamonds represent CBM-Cel7A, orange triangles represent CBM-Cel6A, red circles represent DCBM-12, green triangles represent DCBM-24, and blue squares represent DCBM-48.

Representation of the B_{max}-range used for free energy calculations

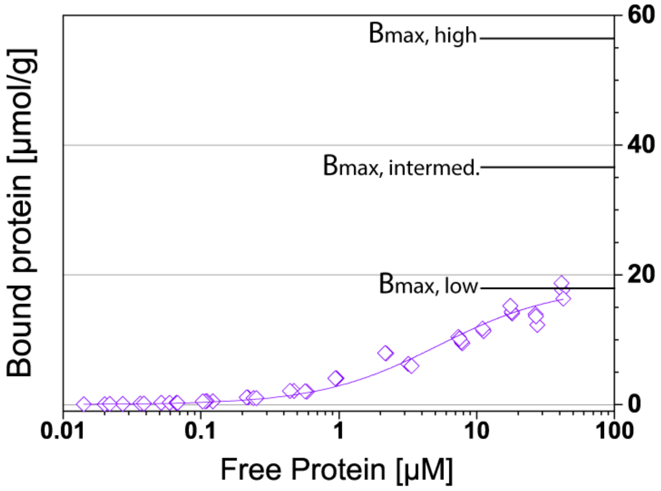

Supplementary figure S3. An illustration of the B_{max}-value range used in the free energy calculations shown on the semi-logarithmic plot of CBM-Cel7A binding isotherm on CNF. The values were used to calculate the range of free energies of binding for CBM-Cel7A, CBM-Cel6A, DCBM-12, and DCBM-24. $B_{max,low}$ represents the experimentally obtained B_{max}-value. The value is lower than in reality and thus represents a minimum which is always exeeded when the cellulose surface is fully covered. $B_{max,intermed.}$ is double that of $B_{max,low}$ and $B_{max,high}$ is thriple that of $B_{max,low}$.

Binding isotherms of CBM and DCBM on pulp

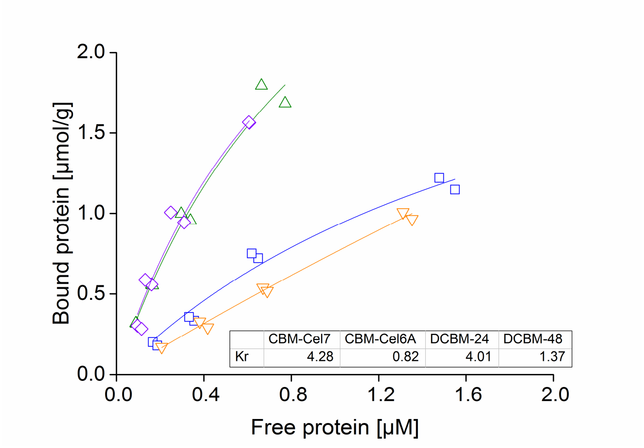

Supplementary figure S4. Binding isotherms of CBM-Cel7A, CBM-Cel6A, DCBM-24, and DCBM-48 on pulp. The partitioning coefficients, K_r, for the proteins are shown in the table within the figure. Violet diamonds represent CBM-Cel7A, orange triangles represent CBM-Cel6A, green triangles represent DCBM-24, and blue squares represent DCBM-48.

Binding affinities and capacities of CBM and DCBM on BMCC and CNF from the initial slope of the binding isotherms

Supplementary Table S5. Values for k_d (µM) and B_{max} (µmolg^{-1}) obtained from curve fitting using binding data presented in Figure 1 and used for calculating K_r values in Table 1.

		CBM-Cel7A	CBM-Cel6A	DCBM-12	DCBM-24	DCBM-48
BMCC	B_{max}	4.26 ± 0.62	5.15 ± 1.81	7.48 ± 0.78	7.07 ± 0.52	3.19 ± 0.20
	k_d	3.03 ± 0.54	8.38 ± 3.31	3.03 ± 0.36	2.51 ± 0.22	2.97 ± 0.24
CNF	B_{max}	28.96 ± 1.42	18.45 ± 2.69	26.22 ± 2.19	29.36 ± 2.69	13.8 ± 4.24
	k_d	5.81 ± 0.37	17.58 ± 3.05	6.77 ± 0.74	7.02 ± 0.79	8.31 ± 3.06

Competition of CBM-Cel7A and CBM-Cel6A on CNF and BMCC

To examine the possible competition of the CBMs on binding sites on BMCC and CNF, we compared how the binding of ^{3}H-labelled CBM-Cel7A was affected by non-labelled CBM-Cel6A and vice versa. A control experiment with buffer only showed the behavior of the protein when the free protein concentration is diluted with no CBM in the solution. This was done in order to see if the added non-labelled CBM affects the binding at all. From the results it was evident that the added non-labelled CBMs affect the binding and they compete with the labelled counterpart in the solution because the results are different form the buffer dilution control. There seems not to be a difference on either substrate whether the competing counterpart is the same or the other CBM because the experiments give very similar results regardless of the components of the experiments. These results suggest that the CBM-Cel7A and CBM-Cel6A fully compete on binding sites on both substrates. This is in agreement with the Gibb's free energies associated with the bindings, also very similar for both proteins on both substrates.

A 25µM solution of CBM containing 10% ^{3}H-protein was diluted 1:1 with a 25 µM solution of the same or the other unlabeled CBM. 100µL of these mixture solutions were let to react with 100 µL of CNF (2 gL^{-1}) and BMCC (1.28 gL^{-1}). As controls the original 25µM solutions with 10% labelled protein and 12.5µM solution with 10% labelled protein (prepared by dilution of 1:1 of the 25µM solution) were used.

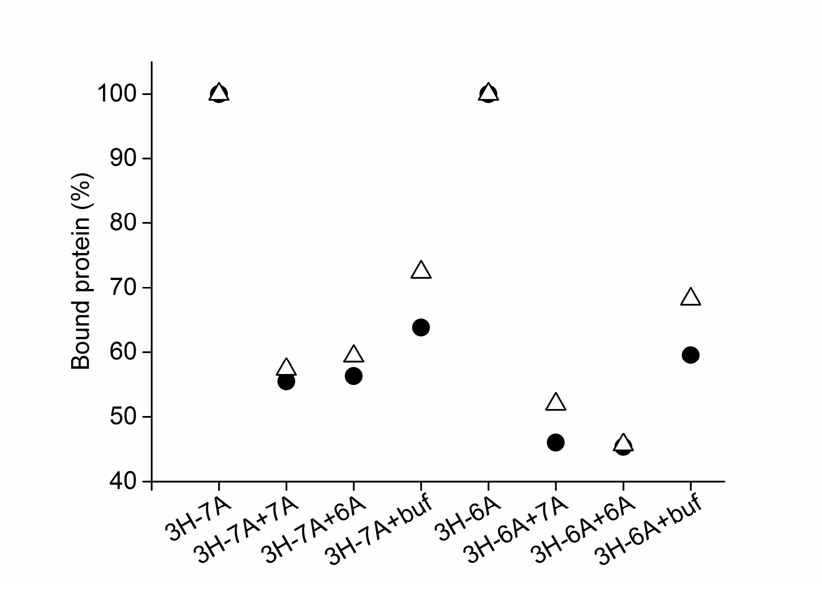

Supplementary figure S6. Competition of ^{3}H-labelled CBM-Cel7A and CBM-Cel6A with non-labelled CBM-Cel7A (3H-7A+7A and 3H-6A+7A, respectively) and CBM-Cel6A (3H-6A+7A and 3H-6A+6A, respectively) on CNF and BMCC. Control experiment with ^{3}H-labelled CBM-Cel7A and CBM-Cel6A without dilution (3H-7A and 3H-6A, respectively), and with 1:1 buffer dilution (3H-7A+buf and 3H-6A+buf, respectively). Δ BMCC, ● CNF.
